# Exploration of nanozymes in viral diagnosis and therapy

**DOI:** 10.1002/EXP.20210086

**Published:** 2022-01-25

**Authors:** Jiyoung Lee, Hongwei Liao, Qiyue Wang, Jieun Han, Jun‐Hyeok Han, Ha Eun Shin, Minghua Ge, Wooram Park, Fangyuan Li

**Affiliations:** ^1^ Institute of Pharmaceutics College of Pharmaceutical Sciences Zhejiang University Hangzhou Zhejiang P. R. China; ^2^ Department of Biomedical‐Chemical Engineering and Biotechnology The Catholic University of Korea Bucheon Gyeonggi Republic of Korea; ^3^ Department of Biotechnology The Catholic University of Korea Bucheon Gyeonggi Republic of Korea; ^4^ Department of Biological Science Korea University Seoul Republic of Korea; ^5^ Hangzhou Institute of Innovative Medicine College of Pharmaceutical Sciences Zhejiang University Hangzhou P. R. China; ^6^ Zhejiang Provincial People's Hospital Hangzhou Hangzhou P. R. China

**Keywords:** antiviral, diagnosis, nanoparticle, nanozyme, treatment

## Abstract

Nanozymes are nanomaterials with similar catalytic activities to natural enzymes. Compared with natural enzymes, they have numerous advantages, including higher physiochemical stability, versatility, and suitability for mass production. In the past decade, the synthesis of nanozymes and their catalytic mechanisms have advanced beyond the simple replacement of natural enzymes, allowing for fascinating applications in various fields such as biosensing and disease treatment. In particular, the exploration of nanozymes as powerful toolkits in diagnostic viral testing and antiviral therapy has attracted growing attention. It can address the great challenges faced by current natural enzyme‐based viral testing technologies, such as high cost and storage difficulties. Therefore, nanozyme can provide a novel nanozyme‐based antiviral therapeutic regime with broader availability and generalizability that are keys to fighting a pandemic such as COVID‐19. Herein, we provide a timely review of the state‐of‐the‐art nanozymes regarding their catalytic activities, as well as a focused discussion on recent research into the use of nanozymes in viral testing and therapy. The remaining challenges and future perspectives will also be outlined. Ultimately, this review will inform readers of the current knowledge of nanozymes and inspire more innovative studies to push forward the frontier of this field.

## Graphical abstract

The various catalytic activities of the nanoparticles and their applications in diagnosing and treating viral diseases and their advantages.

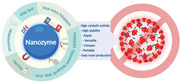

## 1 INTRODUCTION

The efficient and specific catalytic activities of natural enzymes are responsible for numerous biological functions as they can sequentially accelerate important chemical events for the metabolism of life.^[^ ^1^, ^2^ ^]^ The critical biological roles and superior catalytic capabilities of the natural enzymes, such as horseradish peroxidase (HRP) used in enzyme‐linked immunosorbent assay (ELISA), and Taq polymerase applied in a polymerase chain reaction (PCR), enabled them as an ideal candidate for use in biomedicine and industrial catalysis.^[^ ^3^ ^]^ Nevertheless, protein‐originated natural enzymes often fail to meet the needs due to the intrinsic disadvantages, including environment‐induced denaturation or subunit depolymerization‐mediated rapid loss of activity, tedious and long purification processes, difficult storage, and recycling, which severely restricted their usage. Thus, increasing efforts have been devoted to developing novel artificial enzymes that can efficiently function as enzymes.^[^ ^4^, ^5^, ^6^ ^]^

Nanozymes are nanomaterials that share similar catalytic activities with natural enzymes, that is, similar substrates and products, which can catalyze chemical reactions in a way that follows enzymatic kinetics under physiologically relevant conditions, although the underlying molecular mechanisms of nanozymes may to some extent differ from those of corresponding natural enzymes.^[^ ^5^, ^7^ ^]^ Moreover, they are capable of active functioning even under enzyme denaturable conditions. Compared with natural enzymes, nanozymes possess improved physicochemical stability, versatility, and recyclability, which increase their suitability for mass production.^[^ ^8^ ^]^

**FIGURE 1:**
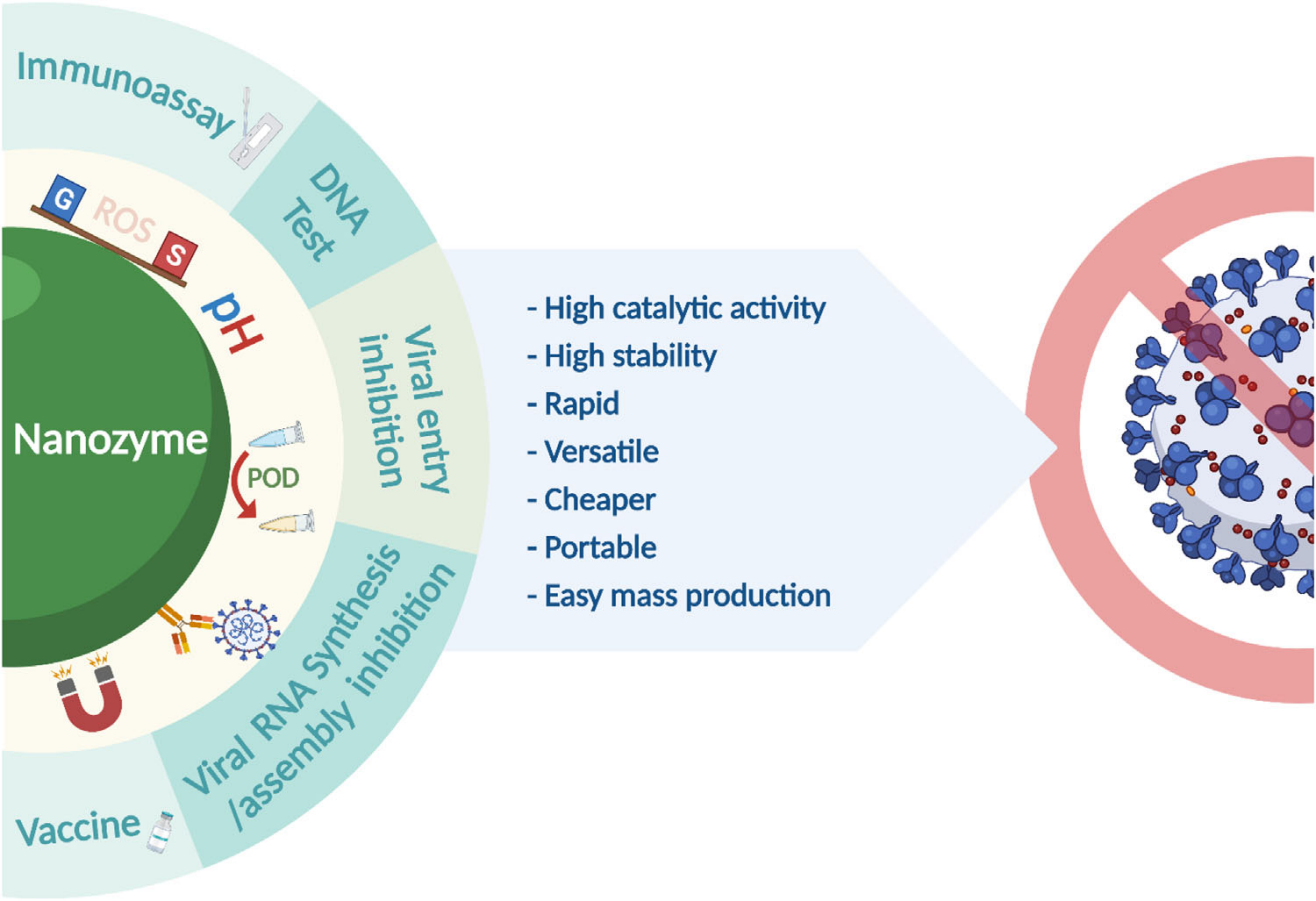
The various applications of nanozymes in diagnosing and treating viral diseases, and their advantages

In the past decade, significant progress has been made in synthesis of oxidoreductase family nanozymes: peroxidase (POD), oxidase (OXD), catalase (CAT), superoxide dismutase (SOD), hydrolase family, and recently lyase family and isomerase family, and in understanding their catalytic mechanisms, which have advanced nanozymes beyond simple replacement of natural enzymes.^[^ ^9^ ^]^ This has tremendously broadened the applications, including biosensing and disease treatment.^[^ ^10^ ^]^ Notably, during the current COVID‐19 pandemic, the exploration of nanozymes as powerful toolkits in diagnostic viral testing and antiviral therapy has attracted growing attention.^[^ ^7^, ^11^ ^]^ Compared with existing viral diagnostic approaches such as the that uses natural enzymes for the visualization, the nanozyme‐based viral testing methods preserve the sensitivity and accuracy, but have much lower cost, mild storage conditions, and can be readily mass‐produced, which are more affordable and may guarantee broader availability that is critical to fighting a rapidly spreading pandemic. Moreover, versatile nanozymes have also been studied for treating viral infection by blocking viral entry, suppressing viral replication, boosting host immunity, and even direct viral destruction, which provides a new weapon in the protracted war against viruses^[^ ^12^ ^]^ (Figure 1).

Herein, we provide a review of the various catalytic activities and representative applications of the nanozymes, with an emphasis on recent exploration of employing nanozymes in viral testing and therapy based on their advantages over natural enzymes. Furthermore, future perspectives will also be discussed in the hope of enhancing the efficacy and clinical translation of nanozymes. Compared with several recently published reviews on similar topics that are restricted to a narrower aspect, for example, a certain virus, or merely application without catalytic mechanisms, our review aims to elaborate on the general principles of nanozyme application in combating virus by connecting different types of nanozymes and their catalytic activities to different stages of management of a broader spectrum of viral infections. Hopefully, this review will deepen the understanding of this promising field by providing a top‐to‐bottom view of the antiviral application‐tailored design of nanozymes, and facilitate the development of more advanced nanozyme‐based antiviral toolkits to push forward the frontier of antiviral research.

## 2 NANOZYME CATALYTIC ACTIVITIES AND THEIR APPLICATIONS

Various enzymes that are involved in complex biocatalytic processes, either individually or collectively, have been identified in biological systems to serve critical roles in the progression of life.^[^ ^2^, ^13^ ^]^ For instance, the innate immune system is capable of producing oxidants in the extracellular fluids and inside the macrophage and neutrophil vesicles.^[^ ^14^ ^]^ During pathogen phagocytosis, a coordinated reaction combines granules containing NADPH oxidase (NOX) and haloperoxidase with halides and subsequently produces site‐specific hypohalous acids (HOXs),^[^ ^15^ ^]^ in which they contribute to the eradication of pathogens encapsulated. NOX converts oxygen to superoxide, which is dismutated spontaneously or enzymatically by SOD to form hydrogen peroxide (H_2_O_2_).^[^ ^16^ ^]^ Haloperoxidases utilize H_2_O_2_ together with pseudohalide thiocyanate (−SCN) or halides to generate HOXs (e.g., hypobromous, hypochlorous, hypoiodous, and hypothiocyanous acids).^[^ ^17^ ^]^ Each of these HOXs is antimicrobial against a diverse array of pathogens, such as bacteria and viruses. Numerous oxidants were believed to eliminate pathogens through oxidization of vital cellular macromolecules.^[^ ^18^ ^]^ Given the essence of natural enzymes in the biological system and their inherent disadvantages, it is crucial to investigate potential alternatives. To date, numerous nanomaterials have been identified as possible enzyme candidates for practical biomedical applications.^[^ ^4^, ^6^ ^]^ For instance, among six different classifications of enzymes, oxidoreductase, hydrolase, lyase, isomerase, ligase, and transferase, we provide a focused discussion on the nanozymes with successfully mimicked catalytic activities, including oxidoreductase and hydrolase with the brief introduction on recently mimicked nanozyme catalytic types such as lyase and isomerase and their applications under the section of others. The remaining ligase and transferase are yet to be mimicked by nanozymes; however, we have highlighted their potential applications, especially in the antiviral treatments in the following section.

### 2.1 Oxidoreductase

Oxidoreductase class of enzymes catalyze an oxidation–reduction reaction, which involves the transfer of electrons from an electron donor, the reductant, to an electron acceptor, the oxidant. Oxidoreductase mimicking nanozymes are the most abundant nanozyme class which are responsible for both reactive oxygen species (ROS) generation and scavenging activities that are often manipulated to control ROS level for diagnosis and disease treatments.

#### 2.1.1 Oxidase

Natural oxidase, an oxidoreductase enzyme subclass, catalyzes the oxidation of a substrate into oxidized products with the aid of oxidizing reagents or molecular oxygen and generates H_2_O or H_2_O_2_. Typically, oxidases are named for their substrates, such as lactate oxidase (LOx), alcohol oxidase (AOx), cholesterol oxidase (COx), glucose oxidase (GOx), and urate oxidase (UOx); the catalytic reactions for oxidases are as follows: AH + O_2_ → A + H_2_O and AH + O_2_ + H_2_O → A + H_2_O_2_. Various nanomaterials have been identified to function as oxidases to date.^[^ ^19^, ^20^ ^]^ Gold (Au), copper (Cu), molybdenum (Mo), palladium (Pd), and platinum (Pt) nanoparticles (NPs) with diameters of 3–5 nm were used to catalyze the glucose oxidation process, with bare AuNPs demonstrating a higher catalytic efficiency.^[^ ^21^ ^]^

Since the identification of AuNPs GOx‐mimicking properties, various gold‐containing bimetallic/trimetallic and supported gold nanoparticles have also been shown to act as GOx mimics.^[^ ^22^ ^]^ Briefly, the gold surface is decorated with hydrated glucose anion, generating negatively charged gold nanoparticles that actively dissolve oxygen to generate a dioxogold intermediate. Ultimately, H_2_O_2_ and gluconic acids were formulated, in which the rate is determined by liquid phase O_2_‐dependent glucose oxidation that initiates glucose to transfer electrons to oxygen.^[^ ^23^ ^]^ Zhang et al. reported Au/Pd nanoclusters (CJ‐Au/Pd NCs) in crown‐jewel like structure. The Pd NCs are decorated with Au atoms on the surface, which promote glucose oxidation.^[^ ^24^ ^]^ This novel crown‐jewel like structure provided high negative charge density to Au, enabling transfer of electron from Au to O_2_, resulting in the generation of hydroperoxo‐like nanocluster. Since the glucose oxidation is highly dependent on active species, it ensures that CJ‐Au/Pd NCs had higher catalytic activity than the monometallic Au, Pd, or even AuPd alloys.

Nanomaterials comprising copper were discovered to be effective oxidase mimics.^[^ ^25^ ^]^ For instance, under the presence of oxygen, fluorescent Cu‐carbon dot nanozymes can oxidize p‐phenylenediamine laccase substrate, which can be applied in the detection of hydroquinone.^[^ ^26^ ^]^ Cytochrome c (Cyt c) donates electrons to cytochrome c oxidase (CcO) to generate a complex with CcO, reducing the oxygen into H_2_O at the heme‐copper center. Cu_2_O NPs demonstrate CcO‐like activity by catalyzing cytochrome c oxidation, leading to the conversion from ferrous to the ferric state.^[^ ^27^ ^]^ Furthermore, laccase is capable of oxidizing a variety of substrates, such as polyamines, polyphenols, and aryl diamines, under the O_2_ supply to produce H_2_O and oxidized products. As the natural laccase contains copper ions as active centers, several Cu‐based nanomaterials were developed to mimic laccase. For instance, Ren et al. synthesized laccase mimicking copper based carbon dots via a one‐pot reaction.^[^ ^26^ ^]^ The 10 nm sized Cu–carbon dots emitted blue fluorescence at 460 nm, and was capable of oxidizing the laccase substrate *p*‐phenylenediamine (PPD) with O_2_.

Furthermore, studies revealed that molybdenum trioxide nanoparticles (MoO_3_ NPs) may act as sulfite oxidase (SuOx) mimic to convert sulfite to sulfate.^[^ ^28^ ^]^ Ultra‐small 2 nm sized MoO_3_ NPs were developed and exhibited high stability in both serum and water. Since SuOx is often found in the mitochondrial membrane and is involved in detoxification, MoO_3_ NP surface was modified with dopamine to anchor the triphenylphosphonium (TPP) ligand for mitochondrial targeting and promote crossing of the membrane. MoO_3_–TPP NPs with such low toxicity may accumulate at the mitochondria and restore SuOx function in SuOx knockdown liver cells, making them potentially useful as therapeutic nanomaterials. In addition, ferroxidases are required for the transport and storage of iron in cellular settings. Recently, several studies have reported ferroxidase‐mimicking PtNPs that can oxidize ferrous ions to ferric ions. For instance, Liu et al. synthesized PtNPs using light‐chain apoferritin as a scaffold.^[^ ^29^ ^]^ This structured nanozyme was capable of regulating cellular iron homeostasis, owing to PtNPs with the ferroxidase mimicking behaviors and the residual ferric ion mineralization capacity of apoferritin. Furthermore, PtNPs synthesized using oligonucleotides demonstrated laccase‐like activity that were capable of oxidizing a variety of laccase substrates (e.g., hydroquinone, catechol, and dopamine).^[^ ^30^ ^]^

#### 2.1.2 Peroxidase

POD refers to a broad set of enzymes that are usually engaged in the decomposition of peroxides, compounds with an R‐O‐O‐R structure. The catalytic reactions for peroxidases are as follows: 2AH + H_2_O_2_ → 2A + 2H_2_O and 2AH + ROOH → 2A + ROH + H_2_O. Yan and co‐workers first discovered that Fe_3_O_4_ magnetic NPs (MNPs) possessed intrinsic POD‐mimicking properties, as they were capable of oxidizing colorless POD substrates, including 3,3′,5,′‐tetramethylbenzidine (TMB), 3,3′‐diaminobenzidine (DAB), and o‐phenylenediamine (OPD) into the corresponding colored products under H_2_O_2_ assistance.^[^ ^31^ ^]^ Following Fe_3_O_4_ MNP discovery, various iron‐based alloys have been utilized to create POD‐mimicking nanozymes. Among which, Prussian blue (PB, [Fe_(III)_Fe_(II)_(CN)^6^]^−^) NPs demonstrated fourfold higher k_cat_ with TMB as a substrate when compared with Fe_3_O_4_ MNPs. Additionally, it has been shown that PB exhibits SOD and CAT‐like activity under various pH settings.^[^ ^32^ ^]^

Carbon is a common nanomaterial that can be used as a peroxidase mimic. In 2010, Song et al. revealed that graphene oxides and single‐walled carbon nanotubes both demonstrate peroxidase‐like properties, with H_2_O_2_, pH, and temperature functions comparable to HRP.^[^ ^33^ ^]^ Therefore, other carbon‐based nanomaterials with peroxidase‐mimicking abilities were investigated, including carbon dots,^[^ ^34^ ^]^ carbon nitride,^[^ ^35^ ^]^ and Fe/N‐doped carbon.^[^ ^36^, ^37^ ^]^ For instance, graphene quantum dots (GQDs) exhibited higher peroxidase‐like activity than graphene oxides with a larger size distribution. Numerous noble metal nanomaterials, including silver,^[^ ^38^ ^]^ gold,^[^ ^39^, ^40^ ^]^ palladium,^[^ ^41^ ^]^ platinum,^[^ ^42^, ^43^ ^]^ and their multi‐metallic nanoparticles,^[^ ^44^ ^]^ have been found to act as peroxidase mimics and are extensively utilized in antimicrobials, biosensors, and treatments. For instance, Ye *et al*. developed Pd–Ir NPs with outstanding peroxidase‐mimicking activity, with a catalytic efficiency almost 28 times that of HRP, and further utilized Pd‐Ir nanozymes to create an ELISA for the disease biomarker identification.^[^ ^45^ ^]^ Also, the human prostate surface antigen sensitivity has reached at the femtogram per mL level.

#### 2.1.3 Catalase

CAT is a typical oxidoreductase enzyme and one of the most important antioxidants present in almost all organisms. The catalytic reaction for catalase is as follows: H_2_O_2_ → O_2_ + H_2_O, in which CAT catalyzes the breakdown of H_2_O_2_ to H_2_O and O_2_, thus protecting the cells from ROS damage. Numerous nanomaterials, including PB, metals, and metal oxides, demonstrated CAT‐mimicking properties.^[^ ^46^, ^47^ ^]^ These nanomaterials typically exhibited CAT‐like activity in addition to other enzyme‐mimicking activity, with temperature or pH determining the dominant enzyme‐mimicking activity. For instance, Pd‐ and Pt‐based NPs exhibited superior catalytic activity under acidic pH conditions, when compared to Ag‐ and Au‐based NPs,^[^ ^48^ ^]^ whereas PB, ZrO_2_, and Co_3_O_4_ NPs function as CAT nanozymes under basic pH conditions.^[^ ^49^ ^]^ Moreover, CeO_2_ NPs demonstrated excellent CAT‐mimicking activity by reducing H_2_O_2_ with Ce^4+^ to produce Ce^3+^ while also generating H^+^ and O_2_. The capture of the following H_2_O_2_ molecule would result in the formation of Ce^3+^ together with H^+^ and O_2_, the NPs to the original state.

In addition, at high pH conditions, PB and metal oxide nanomaterials, such as Co_3_O_4_ and ZrO_2_, exhibited catalase‐mimicking properties.^[^ ^50^ ^]^ Mu *et al*. discovered a mild catalase‐like activity while investigating the peroxidase‐mimicking properties of Co_3_O_4_ NPs.^[^ ^51^ ^]^ Additionally, it was reported that altering the pH from low ph to neutral or even high pH conditions improved the catalase‐mimicking capabilities. As PB exists in many redox states and at high pH it has low H_2_O_2_/O_2_ redox potential, H_2_O_2_ may readily promote PB oxidization to BG/PY and simultaneously reducing the PY/BG to PB, resulting in the generation of O_2_.^[^ ^32^ ^]^ Hence, additional nanomaterials that possess peroxidase‐like activity may be explored for catalase‐like activities. Moreover, molecular processes should be understood in order to expand their applications.

#### 2.1.4 Superoxide dismutase

SOD is also an essential antioxidant that mitigates the cells from oxidative damage caused by ROS through the catalyzation of their dismutation into O_2_ and H_2_O_2_. The catalytic reaction for SOD is as follows: 2O_2_^−^ + 2H^+^ → H_2_O_2_ + O_2_. To circumvent the limits of natural SOD and to fight oxidative stress more effectively, multiple nanoparticles mimicking SOD have been employed.^[^ ^52^ ^]^ For instance, numerous carbon‐based nanomaterials, including systems composed of small carbon clusters (C_60_–C_3_) and C_60_, reduced graphene oxide functionalized with hemin, and hydrophilic carbon clusters successfully mimic SOD activities under various experimental conditions.^[^ ^53^, ^54^ ^]^ The antioxidant behavior was attributed to the enzymatic removal of O_2_^–^. Furthermore, the hydrophilic carbon cluster (HCC) performs as an SOD mimic.^[^ ^54^ ^]^ The HCC was synthesized by reacting nitric acid and sulfuric acid with single‐walled carbon nanotubes. Additional poly(ethylene glycol) (PEG) modification of HCC might aid in increasing its water solubility. PEG–HCC was capable of converting O_2_^−^ into H_2_O_2_ and O_2_, but inert to reactive nitrogen species, such as peroxynitrite (ONOO^−^) and nitric oxide (^.^NO). Due to PEG–HCC planar structure and a large number of unpaired electrons, absorbing electrons from O_2_^−^ was simpler, which increased the efficiency of PEG–HCC. PEG–HCC with such great performance would be extremely promising in treatments.^[^ ^54^, ^55^ ^]^

Nanoceria was one of the first nanomaterials to be reported to have SOD‐like properties, which was ascribed to electron transfer between Ce^3+^ and Ce^4+^ mixed oxidation states.^[^ ^56^, ^57^ ^]^ To date, CeO_2_ NPs have been extensively acknowledged as potential SOD candidates.^[^ ^58^ ^]^ Unlike other trivalent lanthanides, Ce may exist as either Ce^3+^ or Ce^4+^.^[^ ^59^ ^]^ Nanoceria has strong catalytic capabilities due to Ce^3+^ and Ce^4+^ ion presence, as well as the oxygen vacancies. This surprising discovery was universal for cerium oxides of all morphologies and sizes, which simplified the regeneration and regulation of Ce^3+^ and increases its potential for biomedical applications.^[^ ^60^ ^]^ Even though the precise mechanism of cerium oxide superoxide scavenging ability remains unknown, multiple investigations have demonstrated that increasing the Ce^3+^/Ce^4+^ ratio results in increased SOD‐mimicking activity.^[^ ^61^ ^]^ Given the relationship between oxygen vacancies and Ce^3+^, the concurrent decrease in the size of cerium oxide size as it generates additional surface oxygen vacancies was used to ensure a high Ce^3+^ concentration.^[^ ^62^ ^]^ As a result, cerium oxides that are less than 5 nm in size have been intensively investigated as SOD mimics.

Recently, Liu et al. developed melanin nanoparticles (MeNPs) that had multiple free radical scavenging properties.^[^ ^63^ ^]^ MeNPs were produced by reacting ammonia with dopamine hydrochloride in ethanol–water and subsequent PEG functionalization for increased stability. These PEG–MeNPs with a size of ∼120 nm exhibited SOD‐like O_2_^−^ scavenging activity, and have the potential to remove the ^.^OH generated by the Fenton‐type reaction between Cu^+^ and H_2_O_2_. As a result of melanin chelating capacity toward Cu^+^, the pre‐added PEG‐MeNPs prior to H_2_O_2_ may prevent the formation of ^.^OH. Due to the residual functional groups, PEG–MeNPs were also capable of effectively detoxifying ONOO^–^ and NO through nitrosation and nitration.

### 2.2 Hydrolase

Hydrolase, a common yet critical enzyme, catalyzes chemical bond hydrolysis under the presence of water, which fragments larger molecules into smaller ones. Hydrolases are categorized according to the kind of bond they operate on; for instance, nuclease is a nucleic acid hydrolase. The catalytic reaction for hydrolase is as follows: A – B + H_2_O → A – OH + B – H. Numerous nanomaterials have been investigated for their ability to mimic hydrolases.^[^ ^64^ ^]^ Among one of the first nanomaterials to be used as hydrolase mimics, the catalytic monolayer functionalized AuNPs through Au‐S bonds deserve special attention. On the surface of AuNPs, alkanethiol ligands containing zinc ion and 1,4,7‐triazacyclononane (TACN) catalytic complex were assembled. These functionalized AuNPs cleaved 2‐hydroxypropyl p‐nitrophenyl phosphate in a manner similar to RNase.^[^ ^65^ ^]^ In comparison to unassembled TACN‐Zn^2+^ catalytic complex and uncatalyzed reactions, functionalized AuNPs improved the reactions by two and four orders of magnitude, respectively.

Among the nanozymes based on MOFs, Zr‐based nanomaterials are often employed as phosphotriesterase mimics to cleave chemical warfare agent the phosphate ester bonds.^[^ ^66^ ^]^ This is due to the structural similarity between the catalytic sites of phosphotriesterase (Zn–OH–Zn) and MOFs structures in which hydroxyl linkages connect Lewis acidic Zr_(IV)_ centers. MOF UiO family, which is a Zr‐based MOF representative, was synthesized by reacting benzene‐1,4‐dicarboxylate (BDC) with Zr ions and has been extensively investigated for its ability to mimic phosphotriesterase. Katz et al. reported that 400 nm UiO‐66 was capable of catalyzing dimethyl‐4‐nitrophenyl phosphate (DMNP) hydrolysis at 25°C with a half‐life of 45 min.^[^ ^67^ ^]^ Additionally, UiO‐66 was modified with –NH_2_ groups, providing proton donor–acceptor centers to the Zr(IV) centers. In comparison to UiO‐66, UiO‐66‐NH_2_ significantly decreased DMNP half‐life (1 min) and demonstrated a surface TOF increase of about 20‐fold. Furthermore, graphene oxides were also utilized for their hydrolase‐mimicking properties.^[^ ^68^ ^]^ For example, graphene oxide combined with peptide nanofibers has the potential to hydrolyze cellulose. Systematic investigations revealed that this hybrid high polysaccharide hydrolase‐mimicking activity results from the reduced steric hindrance to the substrate, peptide fibril structure, and the synergistic effect of peptide nanofibers and graphene oxide.^[^ ^69^ ^]^ Likewise, short peptide based carbon nanotubes may cleave 4‐nitrophenyl acetate.^[^ ^70^ ^]^

### 2.3 Others

In addition to redox enzymes, oxidoreductase and hydrolase, isomerase can also be mimicked by nanozymes. Isomerase possesses a unique characteristic of converting one molecule from one isomer to the other, in which the bond‐forming and breaking are facilitated by isomerase to achieve intramolecular rearrangements and the reaction is as follows: A‐B → B‐A. Notably, the product of the reaction has the identical molecular formula as substrate; however, they differ by the spatial arrangement of bond connectivity. Recently, Li et al. developed cysteine‐derived carbon dot (CD) novel chiral nanozymes that mimic topoisomerase I and induce enantioselective topological rearrangement of supercoiled DNA.^[^ ^71^ ^]^ Topoisomerases are as critical as nucleases since they are capable of rearranging the topology of double‐helical DNA during genetic processes.^[^ ^72^ ^]^ Topoisomerase I is able to cleave DNA double helix single strand to relax supercoiled DNA and re‐ligate the nicked locations, leading to DNA transcription to RNA. It has been demonstrated that the intercalative CDs can catalyze the generation of hydroxyl radicals that cleave the DNA phosphate backbone, thus rearranging the topology of supercoiled DNA.

Furthermore, nanozymes have been reported to successfully mimic lyases. Lyases are enzymes that catalyze the dissociation of a carbon atom from another atom such as sulfur, oxygen, or another carbon atom. They are involved in cellular activities such as the citric acid cycle and organic synthesis processes such as the creation of cyanohydrins.^[^ ^73^ ^]^ Chen et al. reported a novel bionic zeolitic imidazolate framework‐8 (ZIF‐8) nanozyme.^[^ ^74^ ^]^ ZIF‐8 has a comparable geometric structure to human carbonic anhydrase II (hCAII) and possesses similar catalytic activity. In comparison to hCAII, the esterase‐mimicking ZIF‐8 nanozyme has a comparable affinity for *p*‐nitrophenyl acetate and promotes acetylthiocholine hydrolysis reaction and CO_2_ hydration. Due to their comparable compositions and spatial architectures, a variety of ZIFs also exhibit intrinsic enzyme‐like activity. Tian et al. developed photolyase‐like activity of porous nanorods of ceria (*PN*‐CeO_2_) for high UV‐formed cyclobutane pyrimidine dimer (CPD) photocleavage selectivity and UV‐induced DNA damage repair.^[^ ^75^ ^]^ *PN*‐CeO_2_ provided photolyase‐like activity by increasing visible‐light absorption, allowing efficient interaction between the catalysts and CPDs, and then inducing the selective CPD photocleavage into monomers. Additionally, both in vitro and in vivo studies demonstrated its great potential as a DNA photolyase substitute.

Although nanotechnology has successfully developed nanozymes with most of the existing catalytic types, ligase and transferase have not been reported. Nevertheless, they are potential candidates especially in antiviral theranostics that are worth mimicking with nanomaterials. For example, ligase is an enzyme that catalyzes the bonding of two substrates while hydrolyzing ATP. Zhang et al. reported that RING‐domain E3 ligases (RING E3s) are a subfamily of E3 ligases that include one or two RING finger domains. They are involved in a variety of biological activities, including apoptosis, cell proliferation, and immune regulation.^[^ ^76^ ^]^ Numerous RING E3s have been implicated in the host in inhibiting viral replication by modulating immune responses, such as the activation and inhibition of DNA receptor signaling pathways, toll‐like receptors, and retinoic acid‐inducible gene I‐like receptors, as well as modulating major histocompatibility complex cell‐surface expression and co‐stimulatory molecules. According to recent findings, the RING E3s offer new associations between the host and viruses. Likewise, transferase catalyzes functional group transfer from the coenzyme, the donor molecule, to the other accepting molecule. Lindquist et al. discovered that respiratory syncytial virus replicated in viral replication factories (RFs) that interact with stress granules (SGs).^[^ ^77^ ^]^ Isolation of the O‐linked N‐acetylglucosamine transferase (OGT), an enzyme implicated in SG regulation, as well as the 5′ extragenic trailer sequence of the RSV genome, has been reported to be associated with SG suppression, which influences viral replication.^[^ ^78^ ^]^ Therefore, development of the nanozymes that can mimic ligase and transferase may further facilitate the antiviral researches.

In order to enhance the potential of nanozyme to natural enzymes, researchers should focus on engineering the selectivity and activity of nanozymes. Thus far, the majority of research are focused on activity regulation, with just a few studies examining selectivity. Numerous significant factors, such as size,^[^ ^79^ ^]^ composition,^[^ ^47^, ^80^ ^]^ shape and morphology,^[^ ^81^, ^82^ ^]^ surface coating and modification,^[^ ^40^, ^43^, ^83^ ^]^ pH and temperature,^[^ ^84^ ^]^ and light,^[^ ^85^ ^]^ may affect the diagnostic or therapeutic ability of nanozyme against viruses. Owing to the fact that smaller nanomaterials exhibit increased active sites due to increase in the surface‐to‐volume ratio, the majority of research have indicated that smaller nanomaterials exhibit increased catalytic activity.^[^ ^79^ ^]^ Moreover, several properties may manifest themselves only when the size is reduced to a particular amount. For instance, Ce^3+^ is beneficial for nanoceria's SOD‐mimicking properties due to its stability when the size is smaller than 5 nm.^[^ ^56^, ^62^ ^]^ In addition, a cost‐effective and efficient approach to control nanozyme activity is to develop nanomaterial with increased activity or to dope another element. An extensively investigated method was to grow nanoparticle with lower activity, such as Ag and Au, on top of more active nanomaterials, such as Ir and Pt, which would allow for more efficient usage and boost the enzymatic activity of these noble metals.^[^ ^47^, ^80^ ^]^ Furthermore, it is generally established that the shape and morphology of nanozymes significantly influence their catalytic capabilities.^[^ ^81^, ^82^ ^]^ For instance, Singh et al. examined the GPx‐CAT‐, and SOD‐like behavior of various Mn_3_O_4_ nanoparticles, including cubes, flakes, hexagonal plates, nanoflowers, and polyhedra.^[^ ^86^ ^]^ It was reported that nanoflower Mn_3_O_4_ displayed the maximal catalytic activity for all three kinds of reactions, while other shapes demonstrated only SOD‐like activities. Therefore, future studies should also consider these factors to maximize the capacity of nanozyme abilities.

## 3 EXPLORATION OF NANOZYMES IN VIRAL TESTING AND THERAPY

Nanozymes were inspired by nature but are greatly beneficial over natural enzymes owing to high stability, low cost, and ease of production. Their unique physicochemical characteristics provide nanozymes with numerous functions and open up new avenues for future applications. Over the past decade, considerable progress has been made with nanomaterials to mimic novel enzymatic activities by exploring the catalytic processes, controlling the nanozyme activities, and expanding possible applications. Tunable nanozymes may be used to accomplish particular activities such as drug delivery, capturing pathogens, biosensing, minimally invasive surgery, cancer cells isolation, and so on.^[^ ^87^ ^]^ Although many great reviews have been published in the past, the majority of those reviews focused on particular nanozyme themes or were short minireviews.^[^ ^6^, ^7^, ^88^, ^89^ ^]^ In this review, we will instead be focusing on the overview of current research on the use of nanozymes in viral testing and treatment.

### 3.1 Target identification: Nanozyme‐based virus detection

Nanozymes have been developed as an effective tool for viral testing as they can aid in the detection of viruses by examining viral components, such as characteristic proteins and genetic materials, or host antibodies against viral components. Currently, clinical viral testing consists mostly of nucleic acids and plasma antibody assays, which find it hard to reconcile efficiency and accuracy. These methods also require skilled personnel and large equipment that can be rather costly and not readily available or affordable to everyone in need.^[^ ^90^ ^]^ Moreover, the sophisticated sampling procedures require the patients to attend the hospital or gather in designated areas to seek viral tests, increasing the risk of viral exposure. These dilemmas call for a new generation of viral testing utilities that are both efficient and accurate, cheaper to afford, and easier to handle. Nanozymes that have been broadly used in the identification of biological entities from cells to DNA have shown great potential to fill the gap between the practical need and current techniques.^[^ ^91^ ^]^ They can be manipulated to form advanced probes based on fluorescence, chemiluminescence, electrochemical, thermoplasmonic, calorimetry, and surface plasmon resonance, which have been investigated in several pioneering studies on novel viral testing methodologies and will be reviewed in the following subsections.

#### 3.1.1 Principles of virus detection

Virus detection strategies are closely related to the virus structure and its components. In general, each virion is characterized by nucleic acid genomes (RNA or DNA) contained within their capsid protein shell, which is protected by envelop and envelop proteins, where the membranes are acquired from either the cytoplasmic or nuclear membrane of the host cell.

Since the virus‐specific envelope protein, the viral antigen, can be interpreted as the viral presence, protein/antigen detection strategies have been widely used. To make visible or quantifiable detection, “POD” catalytic activity of spontaneous H_2_O_2_ conversion into a product of water and oxygen has been dominated for such detection tools. Coupled with POD, the chromogens, namely TMB, DAB, OPD, are used as electron donors (substrate) for catalyzing peroxides into the water while changing the starting substrate color with respect to the degree of reactivity. The representative detection tools include immunoperoxidase assay, which utilizes the binding of the virus‐specific antibody to the virus, then treats the samples with chromogen solutions for color development. Moreover, the antibody‐bound virus can be exposed to specific wavelength light to visualize the viral presence by detecting the colored antibody.^[^ ^92^ ^]^ The virus protein envelopes can be key to certain virus detection as their protein envelopes can bind to receptors on the red blood cell (RBC) membrane causing hemagglutination, which is then visible under the microscope.^[^ ^93^ ^]^

Besides virus envelop protein tailored detection strategies that are restricted to enveloped viruses, there is a nuclear‐derived detection strategy, which applies to both enveloped and non‐enveloped viruses. For instance, real‐time PCR is a technique that amplifies a minute amount of viral genomes/nucleotide sequence or transcripts from patients within a few hours.^[^ ^94^ ^]^ Viral genome gets labeled during the amplification process and then the fluorescence is measured at the end to determine the viral presence. Furthermore, humoral immunity‐driven virus detection utilizes post‐infection protection achieved by our body, where the virus‐specific antibodies, IgM and IgG, are progressively produced. These antibodies likewise can be detected via assays such as antibody capturing ELISA that the key enzymes play a pivotal role in color development for viral identification with the assistance of substrates.^[^ ^95^ ^]^ These are the common conventional techniques for virus testing.

**FIGURE 2:**
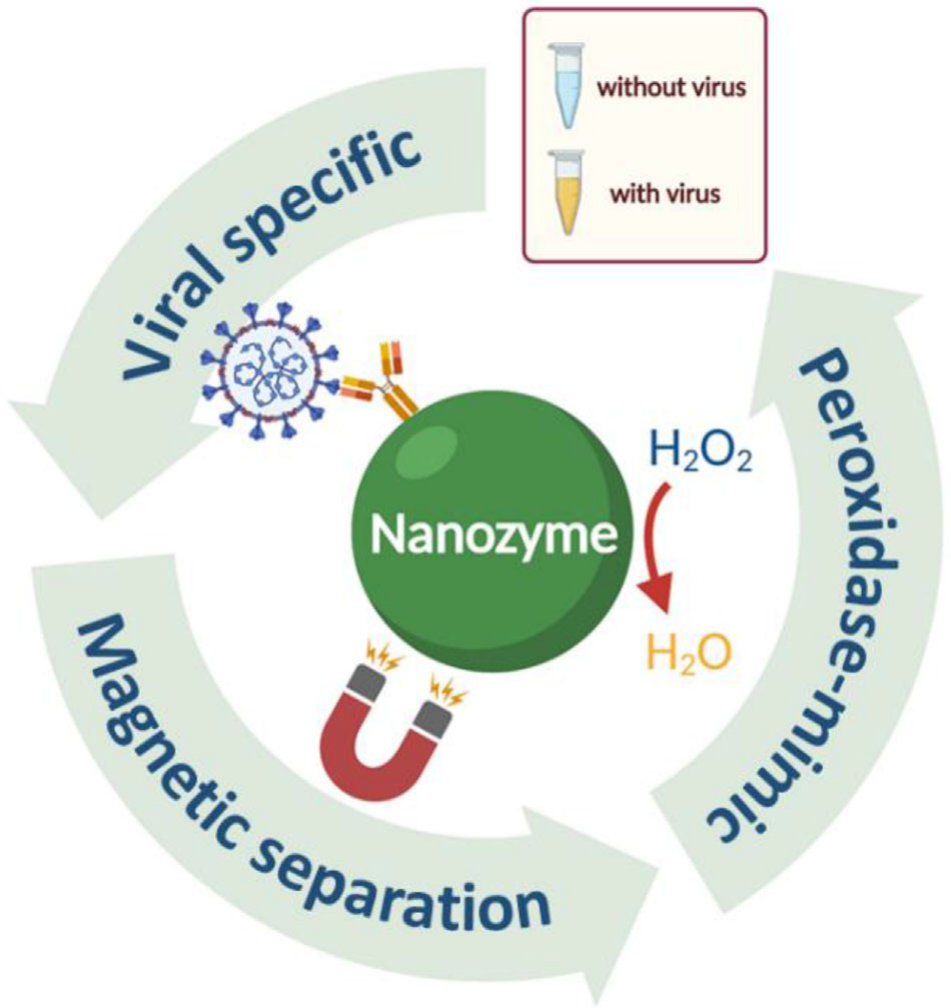
The commonly used nanozyme mechanisms for efficient viral diagnosis

Modern tools have made great advancements to viral detection; however, the enzyme‐based visible and quantifiable viral detections are limited by poor physiochemical stability, immobility, and high cost of production. Therefore, the artificial enzymes (Table 1), the emerging nanozymes, are vital for efficient and effective viral detection (Figure 2).

**TABLE 1:** Nanozyme‐based viral diagnosis

Nanozymes	Activity/Mechanism	Pathogen/Disease	Application	Detection limit	Reference
Tyrosine‐functionalized AuNP	POD	Murine norovirus	Aptasensor for colorimetric detection	200 viruses/mL	^[^ ^97^ ^]^
Fe_3_O_4_	POD	Ebola virus	Immunochromatographic Strip	1 ng/mL	^[^ ^98^ ^]^
Au NP	POD	Influenza A virus	Immunosorbent Assay	44.2 fg/ml	^[^ ^100^ ^]^
Au@Pt NRs	POD	Rubella virus	Immunoassay	10 ng/mL	^[^ ^101^ ^]^
Pt@Au NPs	POD	ZIKV	Specific point‐of‐care (POC) (signal probe)	1 pg/mL	^[^ ^102^ ^]^
CuNFs	POD	Influenza virus	Immunocomplex‐based sending for colorimetric detection	54.97 fg/ mL	^[^ ^103^ ^]^
Co‐Fe@hemin‐peroxidase	POD	SARS‐CoV‐2	Nanozyme chemiluminescence paper test colorimetric detection	0.1 ng/mL	^[^ ^104^ ^]^
NH_2_‐MIL‐101 MOF	POD	Influenza A virus	Acid phosphatase activity screening	0.005 U/L	^[^ ^107^ ^]^
NH_2_‐MIL‐101 MOF	POD	Cancer cell	Circulating miRNA detection	0.8 aM	^[^ ^108^ ^]^
CeO_2_	POD	Cancer cell	Colorimetric detection of DNA	NA	^[^ ^110^ ^]^
MoS_2_	POD	HBV DNA	Colorimetric / fluorometric detection	50 pM	^[^ ^111^ ^]^

#### 3.1.2 Nanozyme‐based immunoassay for viral tests

Numerous nanozymes have previously been shown to be comparable to natural enzymes. The discovery and understanding of nanozyme systems expand biomedical applications to develop biosensors and contrast agents to treat a variety of illnesses. Initially, nanozymes were employed in biosensing and colorimetric studies as substitutes for natural enzymes. The novel enzyme mimicking nanomaterials are characterized by the additional intrinsic properties that natural enzymes lack, thus making it an ideal alternative, especially to conventional colorimetric enzyme‐linked immunoassays.^[^ ^96^, ^97^ ^]^ ELISA is one of the conventional biomarker detection techniques, which typically employs HRP for TMB oxidization to initiate color development and aid quantification. Nevertheless, being a protein‐based enzyme, HRP can be used within a narrow range of pH, temperature, and concentration owing to its instability under severe environments and relatively high cost. Hence, nanozymes have become an ideal replacement due to their greater tolerance for pH and temperature variations, increased amount/concentration when required, and in certain instances, improved catalytic efficiency.

**FIGURE 3:**
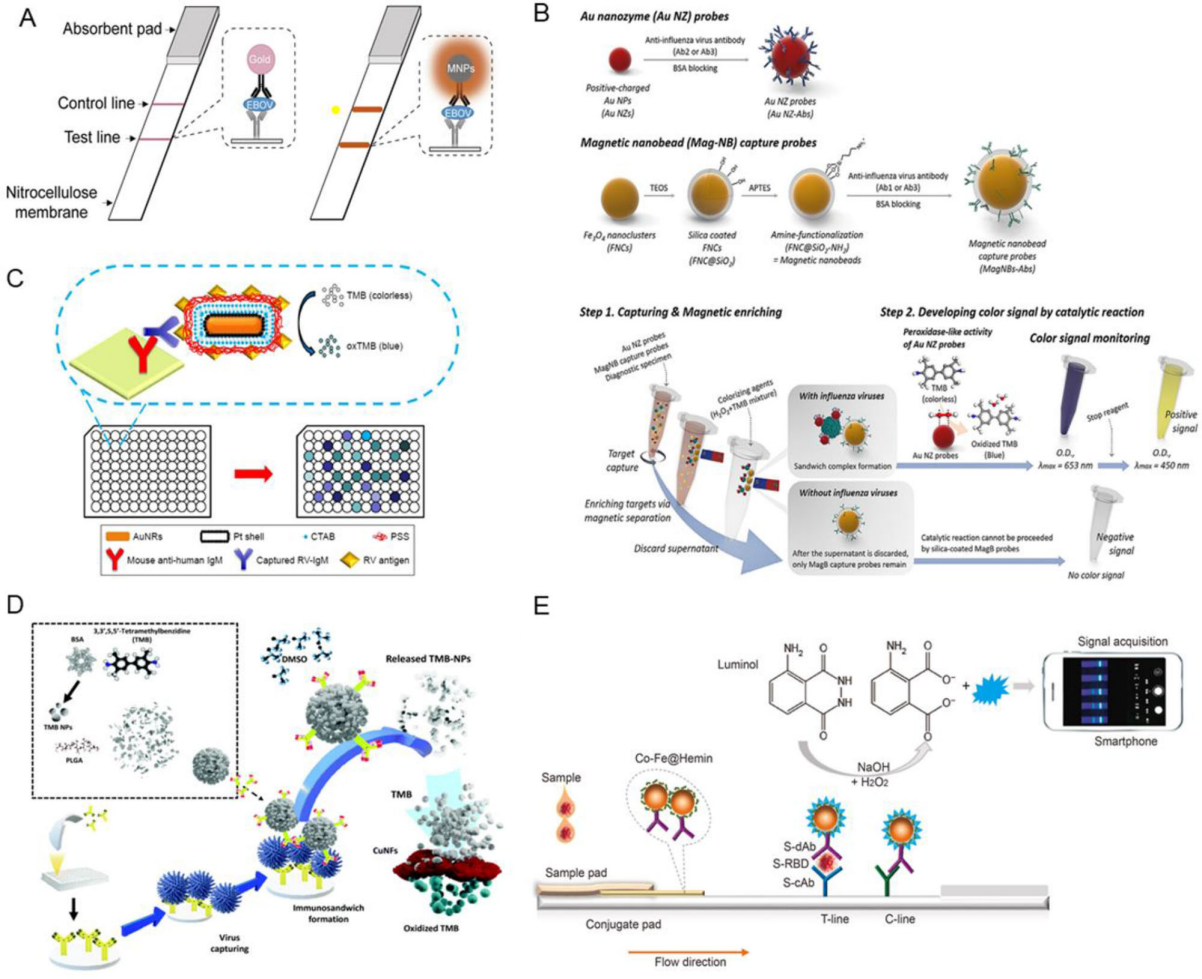
The nanozyme‐based immunoassay for viral tests. (A) The schematic illustration of the conventional colloidal gold strip (left) and Fe_3_O_4_‐dependent nanozyme‐strip (right) for EBOV‐glycoprotein (GP) detection. Reproduced with permission.^[^ ^98^ ^]^ Copyright 2015, Elsevier. (B) The schematic illustration of influenza A virus detection via magnetic nanobead‐based nano(e)zyme‐linked immunosorbent assay (MagLISA). Reproduced with permission.^[^ ^100^ ^]^ Copyright 2018, American Chemical Society. (C) Schematic Illustration of ELISA system based on the Au@Pt NRs conjugated with antigen. Reproduced with permission.^[^ ^101^ ^]^ Copyright 2018, Dove press. (D) The schematic illustration of signal amplification TMB‐NPs@PLGA nanozyme. Reproduced with permission.^[^ ^103^ ^]^ Copyright 2020, Elsevier. (E) The schematic illustration of the nanozyme‐integrated chemiluminescence paper test to detect SARS‐CoV‐2 S‐RBD antigen. Reproduced with permission.^[^ ^104^ ^]^ Copyright 2020, Elsevier

Among various inorganic nanoparticles, iron oxide nanoparticles with excellent POD‐like catalytic activities and magnetic properties promoted advanced biosensor development. Duan et al. fabricated an immunochromatographic strip with the nanozyme, anti‐EBOV antibody labeled Fe_3_O_4_ magnetic nanoparticle (MNP) probe, to detect as low as 1 ng/mL of Ebola virus (EBOV) glycoprotein demonstrating high sensitivity, rapidness, and simpler than ELISA for efficient EBOV screening^[^ ^98^ ^]^ (Figure 3A). In this study, similar to natural enzymes, the prominent POD‐like catalytic activity of Fe_3_O_4_ could catalyze POD substrates to produce a color reaction, which amplified the signal on the strip for improved EBOV glycoprotein detection limit, lowering it by 100‐fold in comparison to that of the standard colloidal gold strip. The magnetic properties of Fe_3_O_4_ enabled rapid separation and enrichment of the target component, which further enhanced the sensitivity by 10‐fold. Additionally, when Fe_3_O_4_ nanoparticles were paired up with corresponding antibodies, they could also successfully detect other diseases, revealing their potential as a simple, rapid, and accurate diagnostic tool with a broad virus detection spectrum. Interestingly, certain metal‐based nanomaterials typically gold nanoparticles (AuNP) exhibited POD‐mimic characteristics that serve as an enzyme mimic for the advanced accurate analytical technique development.^[^ ^99^ ^]^ For instance, Weerathunge et al. developed a colorimetric AuNP nanozyme aptasensor with rapid and sensitive detection of murine norovirus (MNV).^[^ ^97^ ^]^ The POD enzyme‐mimicking activity of tyrosine‐functionalized AuNPs, combined with MNV AG3 aptamer specificity, allows for the production of sensor probes that turns blue under MNV presence with a detection limit of 200 viruses/mL. Similarly, Oh et al. designed magnetic nanozyme conjugated immunosorbent assay (MagLISA), ultrasensitive colorimetric assay consisting of gold nanoparticle and silica‐coated magnetic nanobeads (MagNB) to detect influenza A virus concentrations as low as 44.2 fg/ml^[^ ^100^ ^]^ (Figure 3B). In this study, gold nanoparticles were the key nanozyme to perform POD‐like activity mediated color signal development, which were significantly more potent than the typical biological enzymes. The gold nanozyme conjugated MagNBs is a cluster of < 30 nm Fe_3_O_4_ nanoparticles that exhibited increased magnetization with sustained superparamagnetic property, which promoted magnetic separation, the enrichment of the biomarkers. This probe was further decorated with an anti‐hemagglutinin monoclonal antibody for specific binding to the influenza A virus. Thus, MagLISA demonstrated detection limit of naked human eyes is 5.0 × 10^−12^ g·mL^−1^ but with microplate reader is 44.2 × 10^−15^ g·mL^−1^, a new record for influenza virus screening via enzyme‐linked immunosorbent assay‐based technology.

Moreover, several innovative researchers have combined gold with another metal for enhanced virus detection.^[^ ^101^, ^102^ ^]^ Zhang et al. conjugated antibodies on rod‐shaped platinum‐coated gold (Au@Pt NRs) nanozyme probe revealing active POD‐like activities for rubella virus diagnosis^[^ ^101^ ^]^ (Figure 3C). Notably, the POD‐like activity of the Pt for color signal production has been improved via rod‐shaped gold. The antigen‐conjugated Au@Pt NRs sustained POD‐like activity at a temperature ranging from 25°C to 85°C and pH values ranging from pH 3 to pH 9. Also, they have shown 1000 times more sensitivity than commercial ELISA as the obtained detection limit was 10 ng/mL. Hence, the antigen‐conjugated Au@Pt NRs demonstrate more stability, robustness, and less cost than antibody‐conjugated HRP. Thus, it can be an ideal substitute. The improved catalytic capabilities of this nanozyme probe resulted in a high sensitivity of the antigen‐conjugated Au@Pt NR‐based enzyme‐linked immunosorbent test.

Nanozymes other than AuNP have also been established for viral immunoassay detection. Inspired by efficient antibody conjugation property of carboxylic group rich poly(lactide‐*co*‐glycolide) (PLGA), Khoris et al. engineered PLGA‐based nanocarrier loaded with chromogen substrate, TMB (TMB‐NPs@PLGA), and conjugated with virus‐specific antibodies, aiming to concentrate signal molecules within a single nanocarrier and attain amplified signal upon virus detection^[^ ^103^ ^]^ (Figure 3D). The TMB‐NPs@PLGA nanocarrier formed a specific antibody‐conjugated immunocomplex sandwich structure upon incubation with the influenza virus. When dimethyl sulfoxide is added, the loaded TMB‐NPs are released and actively oxidized by the added substrate solution containing H_2_O_2_ and copper nanoflower nanozyme (CuNF), which exhibits POD‐like activity. Consequently, the solution turns blue as an indication of viral presence. The released TMB molecules demonstrated high detection specificity even at low virus concentrations as low as 32.37 and 54.97 fg mL^−1^ in buffer and serum, respectively.

Notably, nanozymes have also been employed for the detection of COVID‐19. Liu et al. innovatively utilized nanozyme to design portable chemiluminescence paper tests for fast and efficient SARS‐CoV‐2 spike antigen detection^[^ ^104^ ^]^ (Figure 3E). In this study, Co–Fe@hemin‐POD nanozyme was utilized in the paper test based on the concept of a double‐antibody sandwich lateral flow immunoassay. The S‐RBD antibody (S‐dAb)‐labeled Co–Fe@hemin nanozyme chemiluminescence probes are conjugated to a receptor‐binding domain of the SARS‐CoV‐2 spike protein (S‐RBD) and capture antibody of S‐RBD (S‐cAb) to form sandwich immunocomplexes. Next, POD‐like nanozyme catalyzes luminol substrate under H_2_O_2_ presence in alkaline conditions, resulting in chemiluminescence signal generation, which is captured for further analysis. Notably, upon TMB chromogenic reaction, Co–Fe@hemin exhibited a higher catalytic activity of 69.915 U/mg than Co–Fe NPs and Fe_3_O_4_ NPs with catalytic activities of 9.836 and 5.40 U/mg, respectively. SARS‐CoV‐2 recombinant spike antigen has a detection limit of 0.1 ng/mL and a linear range of 0.2–100 ng/mL, demonstrating that it is comparable to natural POD HRP. Likewise, the pseudovirus detection sensitivity was 360 TCID_50_/mL, similar to ELISA. This novel paper test establishes a very sensitive point‐of‐care test platform detecting SARS‐CoV‐2 antigens, which could substantially improve the early identification of SARS‐CoV‐2 infections and significantly reduce the cost (Figure 3J). Collectively, the efficient and portable nanozyme‐based immunoassays have much potential in easy and fast virus diagnosis.

#### 3.1.3 Nanozyme‐based DNA test for viral infection diagnosis

The revelation of nanomaterial enzyme‐mimicking properties has led nanozymes to be prominent research frontiers in various fields, including biosensors. Since DNA aptamer can be the major factor in molecular recognition and function as adhesive substrates to biosensors development, the concept of combining nanozyme applications with DNA (DNA‐nanozyme) is highly appealing for creation of biosensors developed with nanozymes. To date, numerous sensors based on templated or DNA‐functionalized have been identified to detect various targets, thus significantly accelerating the development of nanozyme‐based biosensor.

**FIGURE 4:**
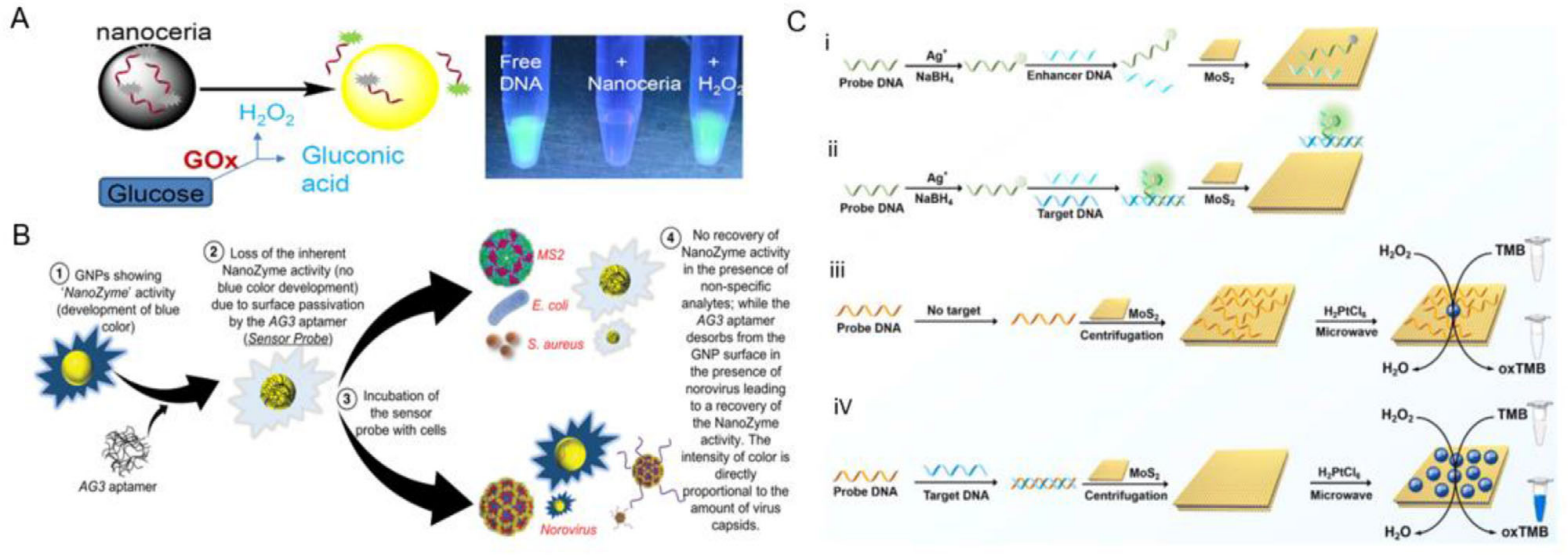
The nanozyme‐based DNA test for viral infection diagnosis. (A) Schematic illustration of NH2‐MIL‐101 MOFs for influenza A virus detection. Reproduced with permission.^[^ ^107^ ^]^ Copyright 2019, Elsevier. (B) Schematic illustration of nanoceria‐based both glucose and H_2_O_2_ sensing via displacement of adsorbed fluorescent DNA. Reproduced with permission.^[^ ^110^ ^]^ Copyright 2015, American Chemical Society. (C) Schematic illustration of AgNCs‐MoS2‐based assay for label‐free HBV DNA detection (i,ii). Schematic illustration of PtNPs‐MoS2‐based sensitive DNA sensing (iii,iv). Reproduced with permission.^[^ ^111^ ^]^ Copyright 2021 Elsevier

Enzymes are frequently used as oligonucleotide probe labels that serve as biosensor signal tags. As a type of enzyme mimicking nanomaterials, nanozymes may likewise be employed as signal tags for DNA probes by functioning similarly to protein enzymes at higher stability and signal performance and lower cost.^[^ ^105^ ^]^ Thus, nanozymes have attracted extended attention due to their potential application as viral sensors. The nanozymes may be utilized to create nanozyme‐based sensors that include labeled probes. DNA probes labeled with protein enzymes may be used to fabricate nanozyme‐based sensors.^[^ ^106^ ^]^ For example, Li et al. developed a new biosensor to screen acid phosphatase (ACP) activity found in influenza A viruses using bifunctional NH_2_‐MIL‐101 metal‐organic frameworks that served as a biomimetic catalyst and fluorescent indicator^[^ ^107^ ^]^ (Figure 4A). The NH_2_‐MIL‐101 MOF nanozyme performs as a POD‐mimic. It can catalyze o‐phenylenediamine oxidation (OPD) to 2,3‐diaminophenazine (DAP) under H_2_O_2_ presence that emitted fluorescence at 556 nm and therefore quenched its emission through the inner‐filter effect. ACP activity detection is based on fluorescence tuning of the NH_2_‐MIL‐101/OPD/H_2_O_2_ system through the pyrophosphate ion (PPi), in which ACP addition restores activity by hydrolyzing PPi while PPi limits the catalytic ability of NH_2_‐MIL‐101 by binding to the Fe center. A ratiometric luminescence signal (F_556_/F_456_) will be produced when ACP and PPi were added to the NH_2_‐MIL‐101/OPD/H_2_O_2_ system. Therefore, a ratiometric fluorescent sensor for sensitive ACP activity screening and PPi detection may be created. Subsequently, Zhang et al. reported on miRNA detection ratiometric fluorescence sensor using GOx‐labeled hairpin probes based on the previous studies on NH_2_‐MIL‐101 MOF nanozyme.^[^ ^108^ ^]^ When the target miRNA is present, it will initiate the catalyzed hairpin assembly process and open the magnetic bead's hairpin probe. The hairpin probe would then initiate the hybridization chain reaction with two GOx‐labeled hairpin probes and bind them to the magnetic beads. Following magnetic separation, GOx will catalyze glucose oxidation to produce H_2_O_2_, which was then utilized to oxidize OPD to detect ratiometric fluorescence by the NH_2_‐MIL‐101 MOF nanozyme. Furthermore, the combination of signal molecule‐labeled DNA probes with nanozymes can be utilized to generate sensors.^[^ ^109^ ^]^ Liu et al. revealed H_2_O_2_ can displace the DNA absorbed by CeO_2_, which prompted a comprehensive investigation of the interaction between H_2_O_2_, CeO_2_, and DNA, resulting in the development of a novel sensing platform for glucose and H_2_O_2_^[^ ^110^ ^]^ (Figure 4B). FAM(fluorescein)‐labeled DNA probe swiftly oxidizes Ce^3+^ to Ce^4+^ under H_2_O_2_ presence. Additionally, Ce^4+^ may be capped with H_2_O_2_, leading to FAM label fluorescence recovery and DNA desorption that can be utilized in qualitative H_2_O_2_ detection. In contrast, without H_2_O_2_, the FAM‐labeled DNA probe is absorbed onto CeO_2_ through DNA phosphate group interaction with Ce^3+^ on the CeO_2_ surface. Overall, the fabrication of DNA‐nanozyme interfaces is an excellent means to enhance detection speed, improve convenience by being label‐free, and increase the sensitivity for virus infection diagnosis. Tao et al. designed a metal‐molybdenum disulfide (MoS_2_) dual‐sensing nanohybrid that can detect Hepatitis B virus (HBV) DNA via colorimetric or fluorometric readouts^[^ ^111^ ^]^ (Figure 4C). First, silver nanocluster (AgNC)‐MoS_2_ nanohybrids were used to detect HBV DNA fluorescence. The detection platform was based on the red fluorescent guanine‐rich tail DNA enhancement and green luminescence bifurcated cytosine‐thymine‐rich DNA enhancement, which can detect two HBV DNAs at the same time. Second, HBV DNA targets act as a growth regulator for platinum nanoparticles (PtNPs) on MoS_2_ nanosheets. The POD‐mimicking activity of MoS_2_ is enhanced by PtNP, and the HBV DNA‐induced growth enables the colorimetric detection of HBV DNAs. The target HBV‐DNA‐induced double‐stranded DNA production may inhibit DNA probe adsorption on MoS_2_ nanosheets, thus facilitating the development of PtNPs. As a consequence, the HBV target DNA may control nanozyme catalytic activity, allowing for the development of a nanozyme colorimetric test for the effective detection of HBV DNA.

Nanoparticles have been demonstrated to increase the sensitivity of immunoassays for the detection of proteins and nucleic acids. Its use in conjunction with ELISA allows for simple and cheap diagnostic tool. Metallic nanoparticle ability to act as enzyme mimics is being investigated as a possible substitute for expensive biological enzymes. Additionally, biological enzymes can readily denature under harsh conditions or high temperatures, unlike enzyme‐mimicking metal nanoparticles.^[^ ^84^ ^]^ The larger nanoparticle surface area, as well as the rapid and efficient electronic transfer that occurs on the surface, enhances the assay's sensitivity.^[^ ^40^, ^43^, ^79^, ^82^, ^83^, ^112^ ^]^ The high sensitivity of nanozyme in a diagnostic environment allows them to reliably diagnose diseases under extremely low amounts of serum biomarkers, such as viral biomarkers. Nevertheless, there are still various limitations for nanozymes. For example, environmental variables, such as temperature and light, may alter the nanozyme metal precursors utilized in the assays, leading to false negative or positive viral tests. As a result, systematic investigations must be conducted to increase assay stability for improved diagnostic functions.^[^ ^99^, ^113^ ^]^

### 3.2 Nanozyme‐based antiviral therapies

**FIGURE 5:**
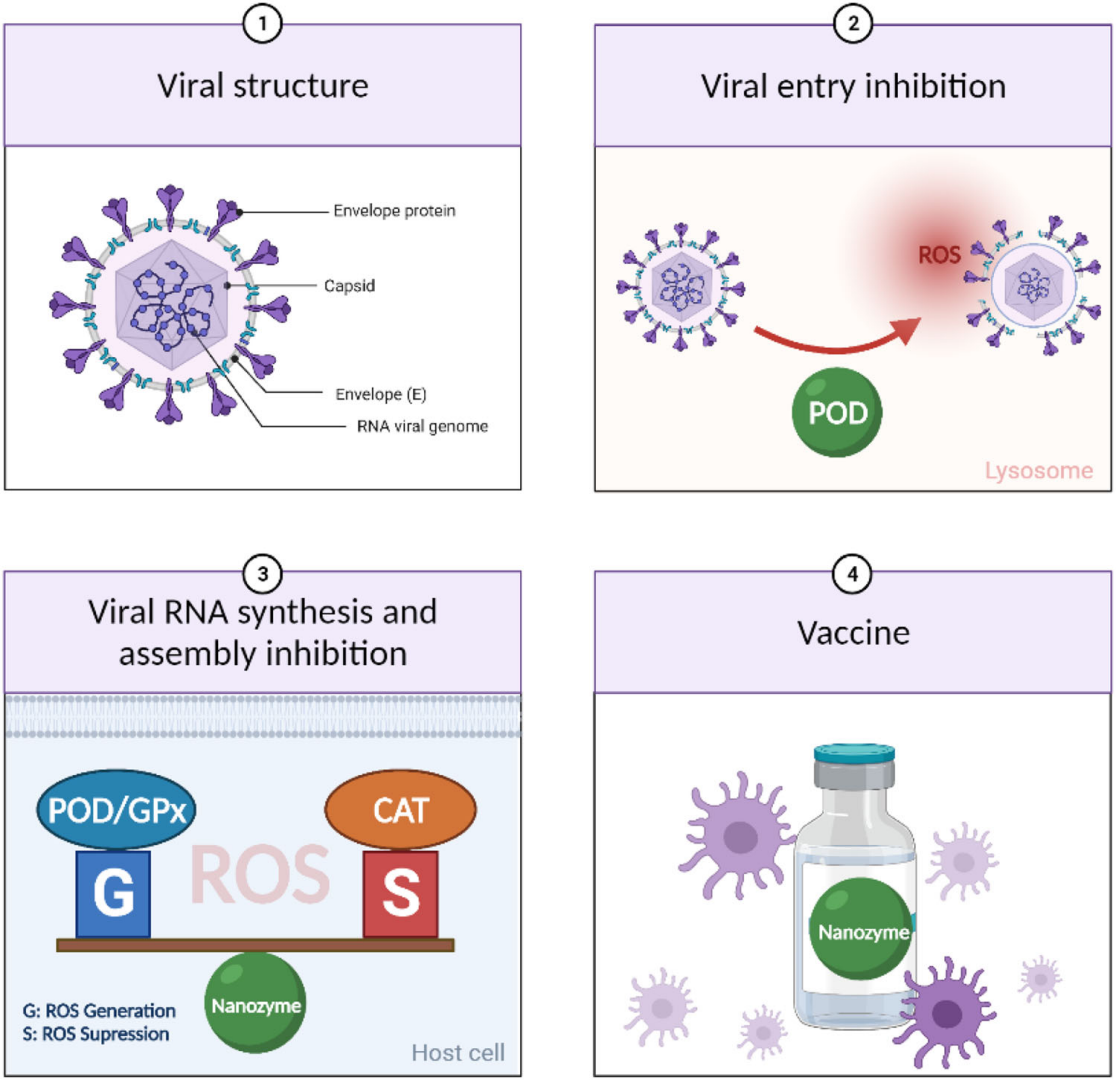
The schematic illustration of virus structure and nanozyme applications for efficient viral treatments

While nanozymes were first identified for their enzyme‐like activity, subsequent studies have focused on the highly‐ordered synergistic nanozyme usage for biological applications. Through the re‐examination of how basic environmental factors may affect nanozyme performance, we can better tailor nanozyme diagnostic and therapeutic roles. Besides the enhanced viral detection, POD‐like, glutathione peroxidase (GPx), and CAT‐like catalytic properties of the nanozymes can also control ROS generation/suppression to tackle the virus at different points in time of the viral infection progression or post‐infection for successful antiviral therapies. Viruses require living host cells to replicate,^[^ ^114^ ^]^ and their structure and other distinctive characteristics are associated with their entrance mechanism, which is classified into endocytic and non‐endocytic routes. Hence, a strategy to eliminate viral infection is to inhibit viral entry, where the first contact with the host cell occurs via receptor blocking and or ROS‐mediated structural deformation. Followed by the attachment to the host cells, viruses go through several phases, such as penetration, uncoating, biosynthesis, maturation, and release (Figure 5).^[^ ^20^ ^]^ Therefore, viral RNA synthesis and assembly inhibition as well as other therapeutic strategies, including the nanozyme vaccine, are also critical for successful antiviral treatment. These are the main areas of nanozyme involvement in antiviral treatment that we will describe in the following sections (Table 2).

**TABLE 2:** Nanozyme‐based antiviral therapies

Nanozymes	Activity/Mechanism	Pathogen/Disease	Application	Outcome	Reference
Ag‐TiO_2_ SAN	POD	SARS‐CoV2	ROS‐mediated viral elimination	99.65% SARS‐CoV2 adsorption by Ag‐TiO_2_ SAN	^[^ ^116^ ^]^
Fe_3_O_4_ IONzymes	POD	Influenza A Virus	Lipid peroxidation‐mediated viral inactivation	H5N1 virus treated with IONzyme (4 mg/mL) induced 15.1 ± 0.7 % cells with late apoptosis (similar to uninfected cell)	^[^ ^117^ ^]^
V_2_O_5_ Nanosheets	GPx	HIV	Redox balance disruption/virus transactivation	Less than five gag fold replication change by nanozyme vs. above 15 gag fold change by untreated ones	^[^ ^118^ ^]^
CS‐IONzyme	POD	H1N1	ROS‐dependent DC maturation	100% protection by CS‐IONzyme vs. 30% protection by H1N1 WIV	^[^ ^123^ ^]^
Ag_2_S	POD	PEDV	RNA synthesis suppression	Ag_2_S NCs inhibited infection for a 3.0 log reduction in virus titer	^[^ ^121^ ^]^
Fe_3_O_4_	CAT	Malaria	ROS‐dependent BBB protection	Fenozyme treated brain tissues of the experimental cerebral malaria mice exhibited negligible ROS	^[^ ^125^ ^]^

#### 3.2.1 Viral entry inhibition

**FIGURE 6:**
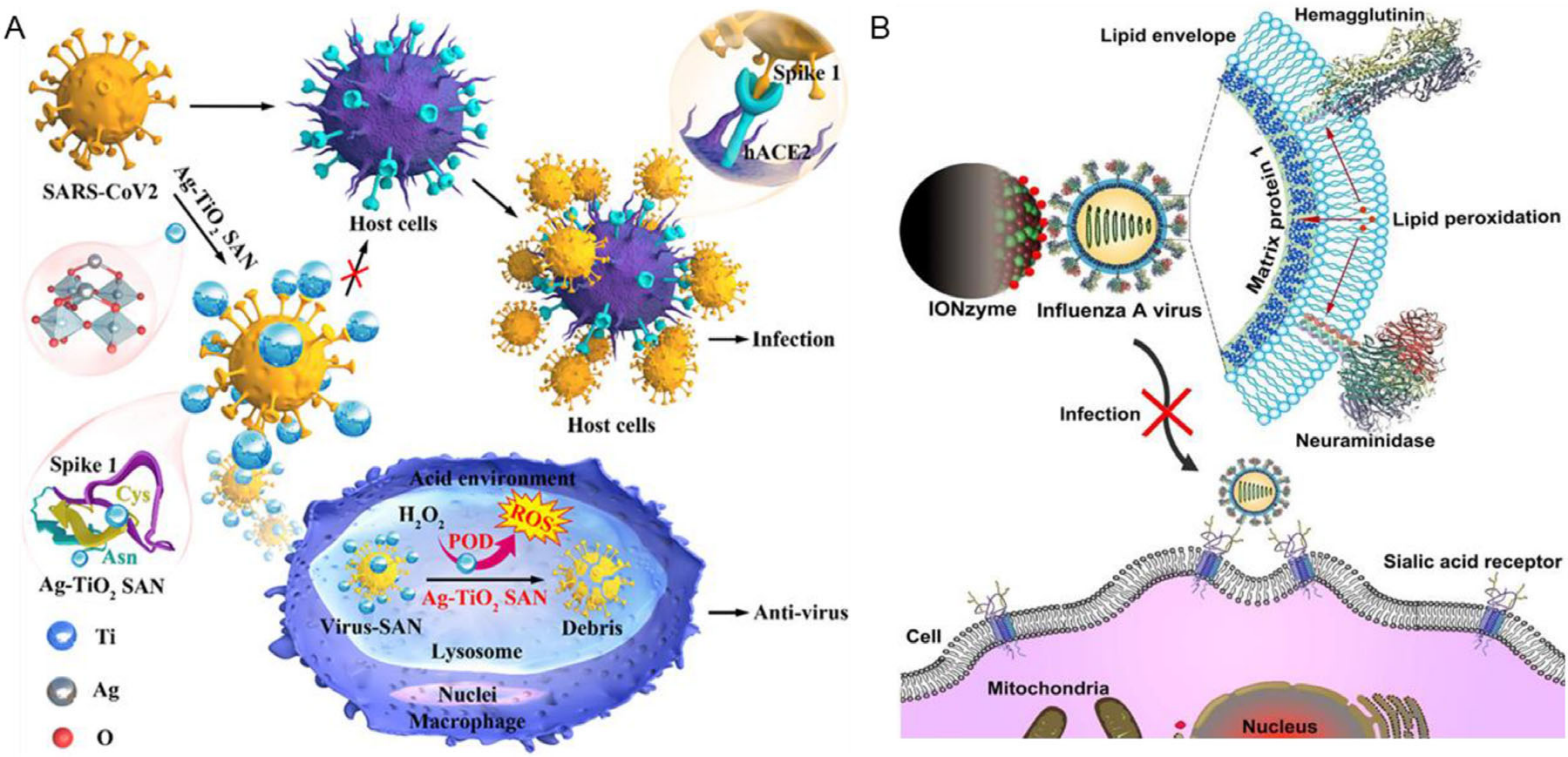
The nanozyme‐based viral entry inhibition strategies. (A) Schematic illustration of Ag‐TiO_2_ SAN demonstrating anti‐SARS‐CoV2 activity via generative ROS within macrophage through POD‐like activity. Reproduced with permission.^[^ ^116^ ^]^ Copyright 2021, Elsevier. (B) Schematic illustration of IONzymes mediated viral lipid peroxidation to inactivate the virus. Reproduced with permission.^[^ ^117^ ^]^ Copyright 2019, Ivyspring

Efficient internalization of the virus by the host cells marks the beginning of successful virus infection as its propagation is highly dependent on the intracellular machinery of the host cells. In such circumstances, inhibiting virus entry to the host cell is crucial for efficient antiviral therapy. As such, interfering with the virus‐host cell interaction by blocking the ligand of the virus is commonly used. For instance, SARS‐CoV‐2, the etiological source of the COVID‐19, is structured in the form of spike (S) proteins with overexpressed ACE2.^[^ ^115^ ^]^ As a central entry gate to the host cell, ACE2 mediates preferential binding to human ACE2 receptors and thus enters the host cell. Hence, Wang et al. engineered a TiO_2_‐assisted single‐atom nanozyme comprising Ag atoms (Ag‐TiO_2_ SAN) to hinder the virus‐ACE2 interaction while exerting enhanced antiviral effects via the POD‐like activity of the Ag‐TiO_2_ SAN^[^ ^116^ ^]^ (Figure 6A). In particular, the silver atoms on SAN rapidly interact with cysteine and asparagine, the common amino acids present on the receptor‐binding domain (RBD) of SARS‐CoV‐2 spike 1 proteins, which in turn averts the host uptake. SAN achieves efficient RBD binding through maximized atomic utilization efficiency as well as the active site density. In which, SARS‐CoV‐2 pseudovirus in vitro absorption was increased by up to 99.65% with SAN. This Ag‐TiO_2_ SAN initiates ROS production via POD‐like activity once it is colocalized in the acidic lysosomes of the macrophage. The acidic lysosome microenvironment improves the oxygen reduction reaction mechanism used to efficiently kill viruses, making it an ideal option for antiviral treatment.

In addition to the blockage of virus‐host cell interactions, disruption of viral lipid membrane is an alternative strategy to effectively avoid viral entry to the host cell. Influenza A virus is a type of virus having a configuration maintained with a layer of the lipid membrane. The multivalent iron oxide nanozymes (IONzymes) with POD‐like activity can actively initiate colorimetric reaction of TMB and H_2_O_2_ while simultaneously inducing lipid peroxidation of the viral lipid envelope, rupturing the envelope structure and impairing the integration of the surface proteins, including hemagglutinin and neuraminidase, and matrix protein 1, which has a transmembrane domain present inside and close to the inside of the lipid layer, respectively^[^ ^117^ ^]^ (Figure 6B). When H5N1 virus incubated with IONzymes were used for TCID_50_ in Madin–Darby canine kidney (MDCK) cells, Log10 TCID_50_ per 0.1 mL was decreased from 4.33 ± 0.00 (untreated) to below the detection limit (treated with 4 mg/mL of IONzymes under 2 h), demonstrating that IONzymes have successfully protected the cells from H5N1 virus infection. Evidently, in the antiviral process, IONzymes exhibit lipoxidase‐like activity, which resulted in this lipid peroxidation‐mediated inactivation of viruses and may aid in the knowledge of antiviral properties of iron oxide and other inorganic nanomaterials. Collectively, IONzymes rapidly catalyzed the viral lipid envelope for the successful prevention of viral transmission and infection. Due to the ease of production and great biocompatibility of IONzymes, it is an intriguing candidate for effective and safe early phase antiviral treatments. As a result, the IONzyme represents a potentially game‐changing strategy to the battle against SARS‐CoV‐2. Thus, multifunctional nanozymes with broad‐spectrum exhibited precise targeting and allowed direct interference of fusion with the virus membrane. The demonstrated significant enhancement of antiviral activities reveals nanozymes as a potential therapeutic candidate in replacing the unsatisfying single usage of virus attachment inhibitors alone and other conventional methods.

#### 3.2.2 Viral RNA synthesis and assembly inhibition

**FIGURE 7:**
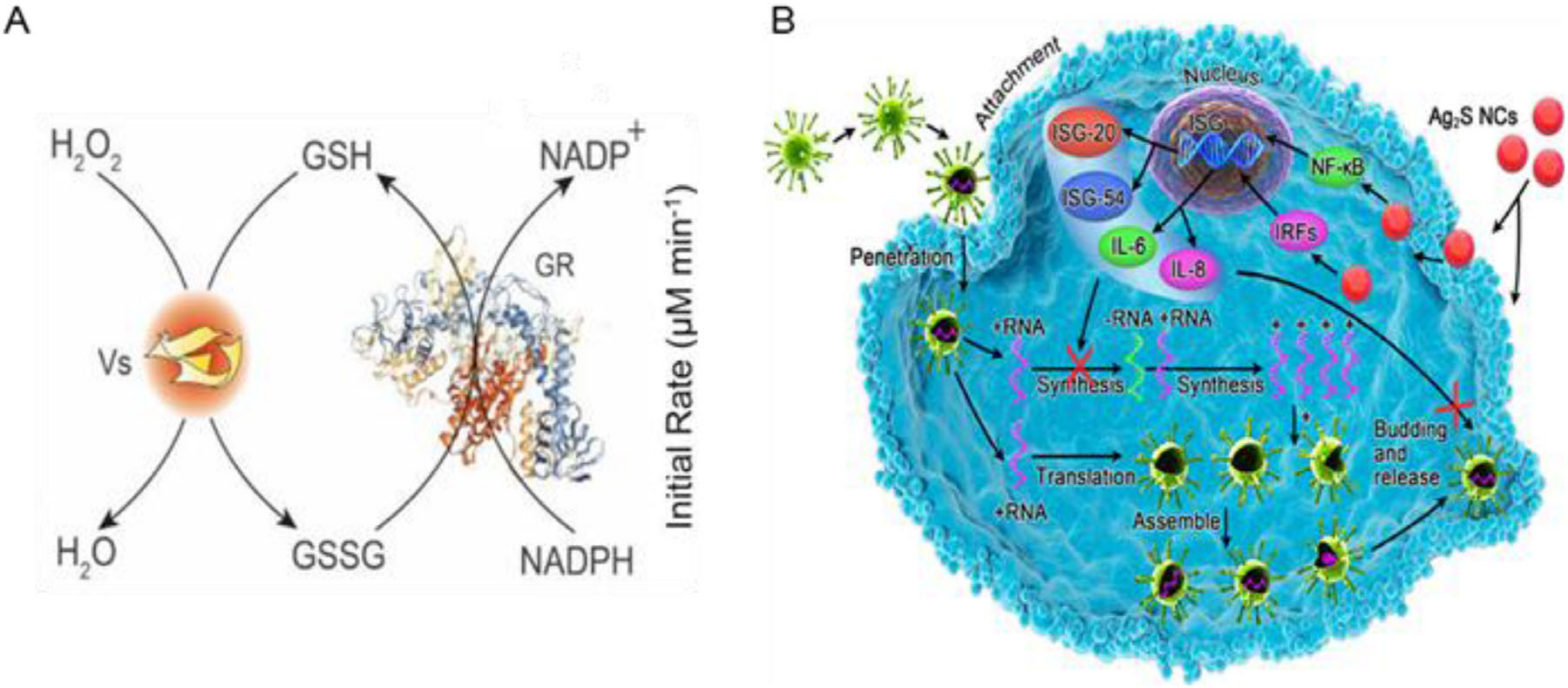
The nanozyme‐base viral RNA synthesis and assembly inhibition strategies. (A) Schematic illustration of GP_X_‐like activity of Vs measurement via glutathione reductase (GR)‐coupled assay. Reproduced with permission.^[^ ^118^ ^]^ Copyright 2021, EMBO Press. (B) Schematic illustration of Ag_2_S NCs antiviral activity by preventing synthesis of viral negative‐strand RNA and viral budding synthesis. Reproduced with permission.^[^ ^121^ ^]^ Copyright 2019, American Chemical Society

Once after viral entry into the host cell, the following immediate yet critical process, viral replication, is the next ideal target for enhanced treatment with novel nanozymes. Therefore, RNA polymerase activity and transcription may be interfered to disrupt the viral replication cycle. These antiviral therapies limit the viral population, allowing the immune system to combat it readily. Singh et al. demonstrated vanadium pentoxide (V_2_O_5_) nanosheets with GPx mimicking activity to reduce ROS‐associated HIV‐1 infection without impairing normal cell function^[^ ^118^ ^]^ (Figure 7A). V_2_O_5_ nanosheets accelerated ROS neutralization in HIV‐1 infected cells and inhibit viral replication and reactivation via genetic reporters for glutathione redox potential and H_2_O_2_ in a mechanistic manner by modulating pathway expression involved in viral transactivation, inflammation, redox balance, and apoptosis. Notably, the combination of V_2_O_5_ nanosheets with NF‐kB inhibitor (BAY11‐7082) completely inhibited HIV‐1 reactivation. V_2_O_5_ nanosheets prevent virus reactivation in HIV‐infected patients with latently infected CD4+ T cells that are undergoing suppressive antiretroviral treatment when stimulated with prostratin. This indicates that nanozymes can be used as future platforms to establish novel infectious disease therapeutic options.

Besides inhibiting genetic material replication or virus‐cell attachment, the alternative method to cease viral infection is virus destruction or inactivation. Due to the nanoscale size of most nanozymes, they may have a high affinity for viral attachment, as they are capable of interacting and directly inactivating viruses without causing damage to host cells.^[^ ^119^ ^]^ Numerous nanoparticles have been studied for their ability to suppress enveloped viruses. For instance, zinc salt was utilized to limit hepatitis E virus replication by inhibiting viral transcription. Nanozymes based on zinc may be tailored to produce Zn^2+^, which could bind to RNA polymerases and suppress SARS‐CoV‐2 RNA transcription. It has been reported that carbon‐based nanozymes have the potential to enhance virus‐nanomaterial interactions.^[^ ^89^ ^]^ Also, a virus inhibitory mechanism using glycyrrhizic acid‐based carbon dots (Gly‐CDs) has been developed for swine pseudorabies virus and reproductive and respiratory syndrome virus, which demonstrated remarkable antiviral efficacy by stimulating IFN production in cells and inhibiting virus invasion, replication, and viral‐infection‐induced ROS generation. Silver nanoparticles and nanowires at concentrations below the hazardous threshold have been used to decrease the infectivity of various coronaviruses by inhibiting RNA synthesis from ceasing viral proliferation.^[^ ^120^ ^]^ Additionally, Du et al. engineered glutathione‐coated Ag_2_S nanoclusters(Ag_2_S NCs) with antiviral treatment properties against the porcine epidemic diarrhea virus (PEDV), in which PEDV infection was inhibited three times after 12 h post‐infection (Figure 7B). Ag_2_S NCs can inhibit viral infection with a 3.0 log reduction in virus titer at 12 hpi, demonstrating successful antiviral activity against PEDV infection at non‐cytotoxic concentrations. The antiviral mechanism of nanoclusters was responsible for the cessation of viral negative‐strand RNA production and budding.^[^ ^121^ ^]^ Moreover, interferon and cytokine production was increased to eliminate coronavirus infection. These findings indicate that viral inactivation involves more than one pathway, and may serve as a guideline for future research on SARS‐CoV‐2. Therefore, silver‐based nanozymes can improve antiviral ability by inhibiting viral entry and replication of SARS‐CoV‐2.

#### 3.2.3 Nanozyme vaccines and others

**FIGURE 8:**
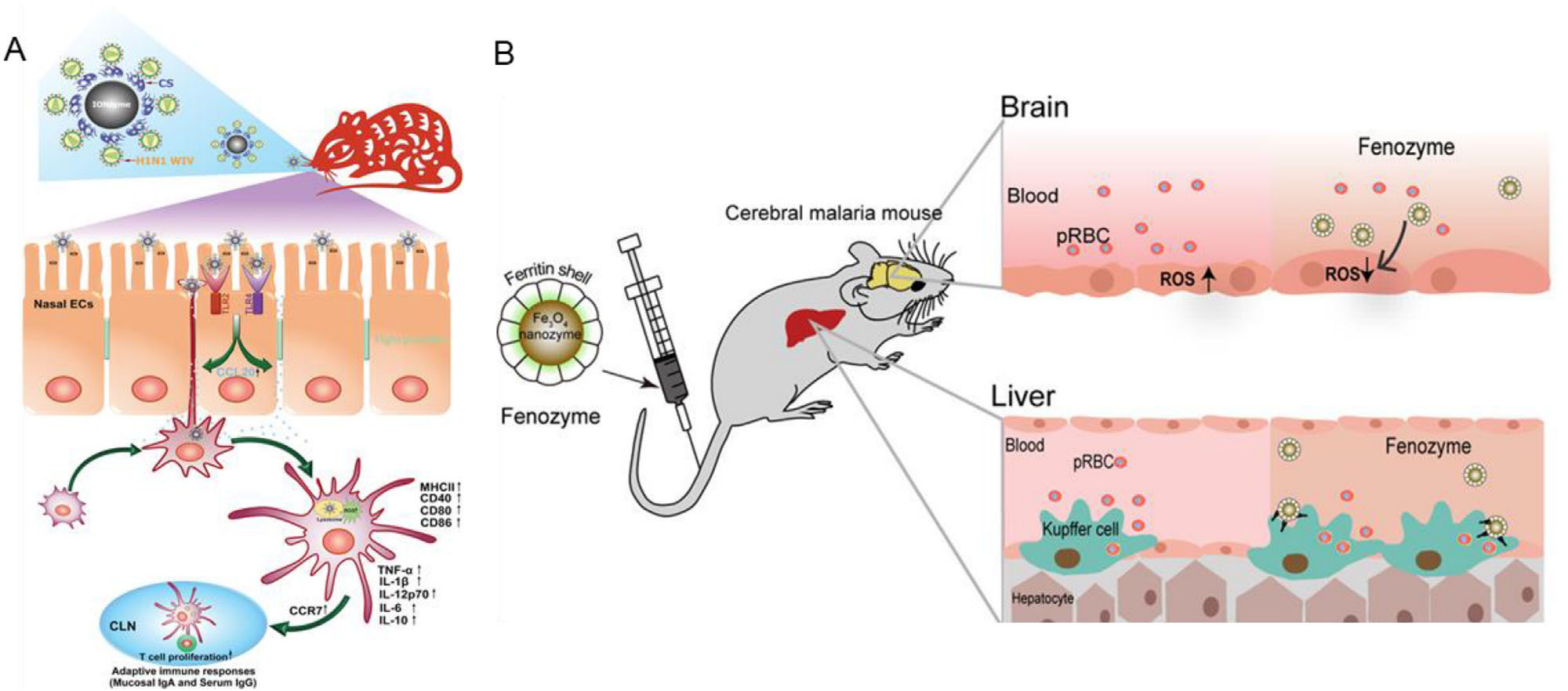
The nanozyme‐based other antiviral therapies. (A) Schematic mechanism illustration of CS‐IONzyme‐based vaccine to enhance the antigen‐specific immune response against influenza. Reproduced with permission.^[^ ^123^ ^]^ Copyright 2020, Wiley. (B) Schematic illustration of fenozyme based protection against cerebral malaria. Reproduced with permission.^[^ ^125^ ^]^ Copyright 2019, American Chemical Society

Vaccine development is an obvious strategy to attain complete control over combating viral infections in the current pandemics.^[^ ^122^ ^]^ Thus far, nanozymes have coordinated innate and adaptive immune systems by protecting proteases and nucleases coupled with adjuvant activity to achieve much‐desired herd immunity and thus virus elimination. Due to the first contact of the virus through the nasal cavity, Qin et al. designed a whole inactivated virus (WIV)‐based nasal vaccine composed of chitosan (CS) decorated with iron oxide nanozyme (CS‐IONzyme) to achieve intranasal immunization^[^ ^123^ ^]^ (Figure 8A). The CS modification altered the IONzyme surface charge from negative to positive charge, which enhanced antigen adhesion to the nasal mucosa by 30‐fold compared to H1N1 WIV alone. Consequently, increased adhesion promoted efficient POD‐like activity of IONzyme that catalyzes ROS‐dependent DC maturation, enhancing H1N1 WIV‐loaded DC migration into the draining lymph nodes for antigen presentation. As a result, the CS‐IONzyme‐based nasal vaccine successfully exhibited increased IgA‐mucosal adaptive immunity by 8.9‐fold, demonstrating complete protection against influenza, compared to H1N1 WIV with 30% insufficient protection. In addition, subunit vaccinations may fail to stimulate a strong CD8^+^ T‐cell immunological response against intracellular infections.^[^ ^112^ ^]^

Intriguingly, nanozymes may also function as immunostimulatory molecules, activating the immune system defense mechanism against SARS‐CoV‐2. Thus, future vaccine development with special nanozyme properties may play a significant role in overcoming the challenges of establishing an effective vaccination and ensuring its global availability. The immune system antiviral mechanism generally involves antibody production, tumor necrosis factor, and interleukins, which are two crucial cytokines for immune responses against viruses. An effective vaccine against viruses is expected to be safe and immunogenic by balancing T and B cell activation for a reliable long‐term immune response.^[^ ^124^ ^]^ Nanomaterial‐based vaccines are preferential because they can defend against proteases and nucleases and be exposed to the immune system. Aside from being structural entities, nanozymes can be used as delivery vehicles to stimulate the immune system and increase the thermostability of the vaccine for further distribution.

Moreover, the ROS governing the property of the nanozyme could eliminate virus‐mediated‐ROS production to prevent disease development. Zhao et al. incorporated ferritin nanozyme (fenozyme) to ameliorate parasite‐infected RBC‐mediated blood–brain barrier (BBB) damage (Figure 8B). In this study, fenozyme is composed of BBB endothelial cell‐specific human ferritin (HFn) protein decorated Fe_3_O_4_, demonstrating CAT‐like activity to efficiently scavenge ROS for cerebral vessel endothelial cell protection, and thus inhibit BBB damage.^[^ ^125^ ^]^ The fenozyme administration further mediated the polarization of liver macrophages to the M1 phenotype, which enhanced malaria elimination from the blood. In addition to the immense potential in versatile nanozyme for advanced vaccine development, the unique characteristics of nanozymes in governing ROS production can be utilized to ameliorate the virus‐mediated biological crisis.

## 4 CONCLUSION AND FUTURE PERSPECTIVES

Despite rapid advancements in healthcare research, there are still formidable challenges in viral diagnosis and treatment, leaving us vulnerable to viruses, the silent and invasive killers. In this battle against viruses, nanotechnology‐potentiated diagnostics and biomedicines are much anticipated due to rapidness, high therapeutic efficacy, and convenience as revealed from previous studies.^[^ ^126^ ^]^ Since the 2007 Fe_3_O_4_ nanoparticle discovery as POD mimics, nanozymes have sparked great interest. Nanozymes have been extensively used in various diagnoses and therapeutic applications due to their numerous benefits as natural enzyme mimic, including high physiochemical stability, excellent durability, ease of production, and low cost. Especially for antiviral theragnostic, nanozymes have been used to develop diagnostic toolkits and applied for therapeutic purposes, including suppression of viral proliferation by inducing long‐lasting immunity to avoid recurrent infection, the prevention of viral invasion‐mediated cellular damage, and the reduction of mutation frequency.^[^ ^127^ ^]^ Hence, in this review, we have summarized the catalytic mechanism and fabrication of nanozymes, as well as the recent progress in viral diagnostic and therapeutic capacities.

Even though nanozymes can overcome various drawbacks associated with traditional diagnostic and therapeutic options of natural enzymes, many challenges remain. For example, despite high specificity and sensitivity for recombinant antigen and pseudovirus, nanozyme‐based antiviral test should still be validated using clinical samples with higher complexity.^[^ ^128^ ^]^ A parallel comparison of this strip test with other commercial kits is still needed to commercialize the nanozyme‐mediated detection strategies. Moreover, the mechanisms driving the enzyme‐mimicking properties of the nanozyme are not completely understood, making de novo “fabrication by design” challenging. Since the reaction conditions are significantly more challenging to regulate on a larger scale, further scale‐up synthesis must be validated; otherwise, problems like batch variances may inevitably impede the process of industrialization. Furthermore, considering the evolving nature of viruses due to their high mutation rates, nanozymes will also have to “evolve” in function and strength to countervail, by optimizing morphology, composition, and surface functionalities that can, for example, promote targeting of the virus with high specificity, and prolong therapeutic protection like host immunity. To this end, it is imperative to continuously mature the nanozymes by comprehensively understanding the structure–function relationship of nanozyme themselves, viral characteristics, and viral infection mechanisms, to broaden their antiviral applications.

To accelerate the clinical applications of nanozymes, it is vital to conduct a thorough evaluation of their advantages and risks. These investigations may include, but are not limited to, the evaluations of pharmacokinetics, clinical toxicity, immunogenicity, and the cellular fate of nanozymes. As the safety of the nanozyme is one of the greatest concerns, dosage control and surface modification may aid in reducing toxicity and increasing specificity, yet the associated effect on the subsequent metabolism and catalytic activity of nanozymes must be considered. The substrate specificity of the nanozymes is still far from natural enzymes with excellent substrate selectivity and catalytic activity. Therefore, a deeper knowledge of the nanozyme catalytic mechanism is crucial to unveil the structure–activity association, which would benefit nanozyme catalytic activity regulation and enhance the substrate specificity of nanozymes for improved theranostics performance. Moreover, there is limited understanding of the mechanisms of antiviral nanozymes, which hinders the efforts to further enhance their therapeutic efficacy. Hence, the antiviral mechanisms of nanozymes should be further investigated, for example, by integrating computational and experimental researches, which will facilitate rational design and synthesis of more advanced antiviral nanozymes.

In addition, only a few catalytic mechanisms of nanozymes were reported so far, while numerous catalytic mechanisms remain unclear. The majority of studies on nanozymes have focused on imitating oxidoreductases, whereas other enzymes, particularly stereoselective nanozymes and isonanozymes, have received less attention. Enzymes are generally classified into six groups in the natural environment based on the type of catalytic activity, including hydrolases, isomerases, ligases, lyases, oxidoreductases, and transferases. There are many enzymes found in living organisms that cooperate and engage in a variety of critical biological processes. For instance, the discovery of ligase‐ and transferase‐mimicking nanozymes as antivirals through viral replication inhibition and immune responses modulation has opened up new possibilities for nanozymes that could be further explored.^[^ ^76^, ^78^ ^]^ Hence, the development of nanozymes that can mimic various enzymatic properties will be essential to uncover the hidden potential of nanozymes in the diagnosis and treatment of viral diseases. Besides, the antiviral mechanism of the nanozymes has not been fully investigated. An intensive and in‐depth research would assist the development of advanced nanozymes for antiviral treatments. Nanozymes have shown great potential in the replacement of existing diagnostic strategies, for example, diagnostic strips; however, up‐scale production of thermal stable nanozyme strategies are compulsory to increase their availability across the country. Also, instead of single usage of nanozymes, innovative combinations of biocompatible nanozymes with therapeutics such as adjuvants may further extend the antiviral applications.^[^ ^100^ ^]^

Nanozymes have shown great potential in bringing us a step closer to overcoming global challenges against emerging viruses.^[^ ^98^, ^99^ ^]^ However, the majority of current research is focused on the active sites of relevant enzymes. The enzyme's protein scaffold mimic, which is critical for the efficiency and selectivity and efficiency of an enzymatic process, has received little attention so far.^[^ ^129^ ^]^ In addition, several enzymes that only function correctly in their original environments (e.g., within the lipid membrane) were rarely reported. Therefore, new strategies and nanotechnological advancement with deepened understanding of diseases would broaden the biomedical applications and enable clinical translation.

## CONFLICT OF INTEREST

The authors declare no conflict of interest.
